# Supplementary material for: Selective photocatalytic conversion of methane into carbon monoxide over zinc-heteropolyacid-titania nanocomposites
Source: Nat Commun. 2019 Feb 11;10:700. doi: 10.1038/s41467-019-08525-2 (PMC6370819; doi:10.1038/s41467-019-08525-2)
Supplement: Supplementary file 1 — Supplementary Information [file 41467_2019_8525_MOESM1_ESM.pdf]

Supplementary Information

Selective photocatalytic conversion of methane into carbon monoxide over zinc-heteropolyacid-titania nanocomposites

Xiang Yu et al

Supplementary Table 1. Catalytic performances for photocatalytic CH_4 conversion measured under irradiation at different spectral ranges.

**Spectral range of irradiation (nm)**	**Formation rate (μmol g^{-1} h^{-1})**		**Irradiance（mW cm^{-2}）**	**Formation rate to power ratio (μmol g^{-1} h^{-1} mW^{-1} cm^{2})**
	CO	CO_2		
>382	11	5.9	94	0.12
280-400	208	48	38	5.5

Reaction conditions: catalyst, 0.1 g; Gas phase pressure, CH_4 0.3 MPa, Air 0.1 MPa; irradiation time, 6h; light source, Hamamatsu LC8-06 Hg-Xe stabilized irradiation lamps with a spectral irradiance in the range 240-600; Cut-off filter :Vis-IR $\lambda > 382$ nm; UV light, $\lambda = 280\text{-}400$ nm.

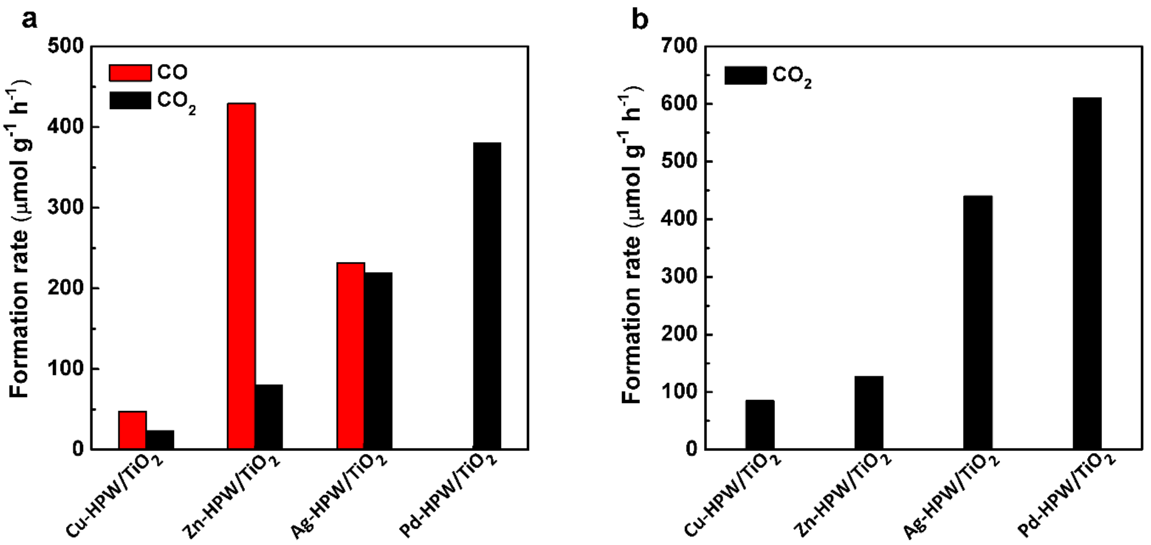

Supplementary Figure 1. (**a**) CO and CO_2 production rates in the CH_4 oxidation and (**b**) CO_2 production rate in the CO oxidation over different catalysts. Reaction conditions: catalyst, 0.1 g; Gas phase pressure, (a) CH_4 0.3 MPa, Air 0.1 MPa; (b) CO 0.3 MPa, Air 0.1 MPa; irradiation time, 6h.

We have conducted a comparative study of both CH_4 and CO photo-oxidation over the HPW-TiO_2 composites containing Cu, Zn, Ag and Pd. The relevant tests were carried out respectively in methane and air (**Supplementary Figure 1a**) and in CO and air (**Supplementary Figure 1b**). Interestingly, the rate of CO_2 production increases in the same sequence (Cu-HPW/TiO_2 < Zn-HPW/TiO_2 < Ag-HPW/TiO_2 < Pd-HPW/TiO_2) in both methane and carbon monoxide oxidation reactions.

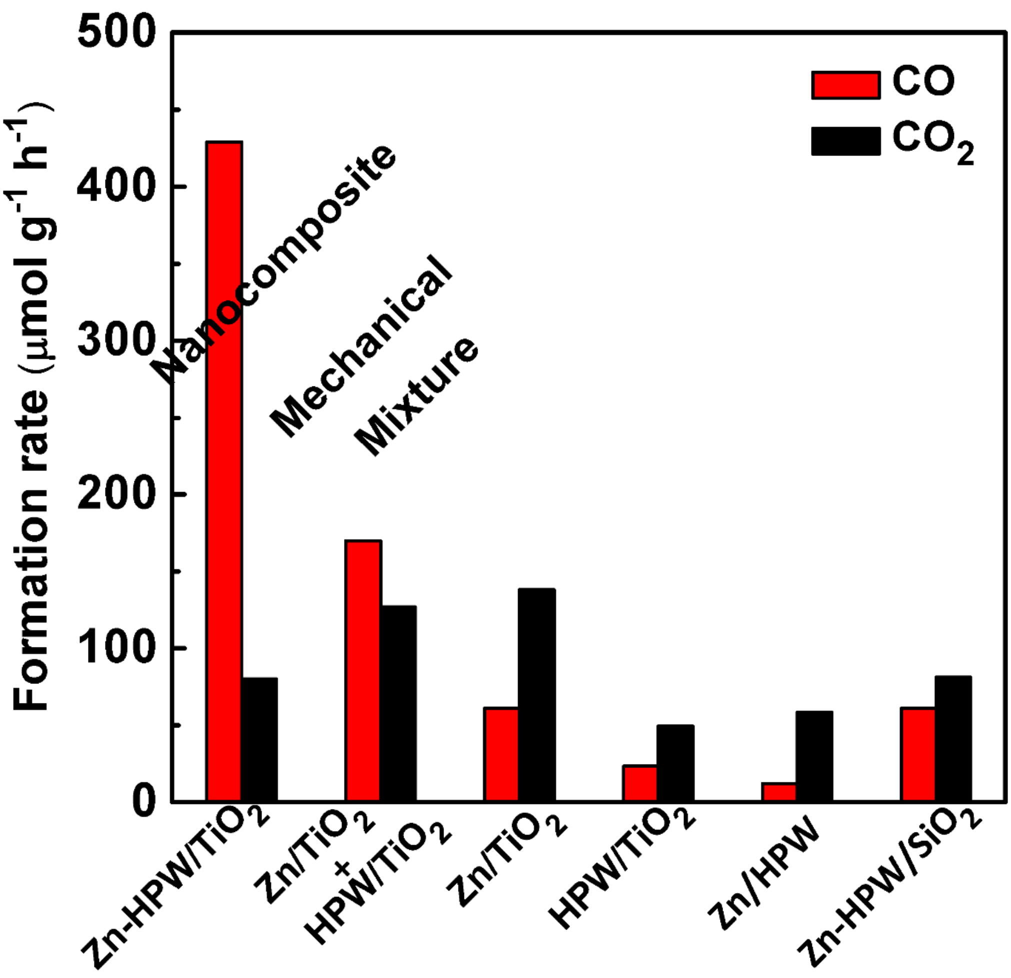

Supplementary Figure 2. Carbon monoxide and carbon dioxide formation rate over nanocomposites and mechanical mixtures. Reaction conditions: catalyst, 0.1 g; Gas phase pressure, CH_4 0.3 MPa, Air 0.1 MPa; irradiation time, 6h.

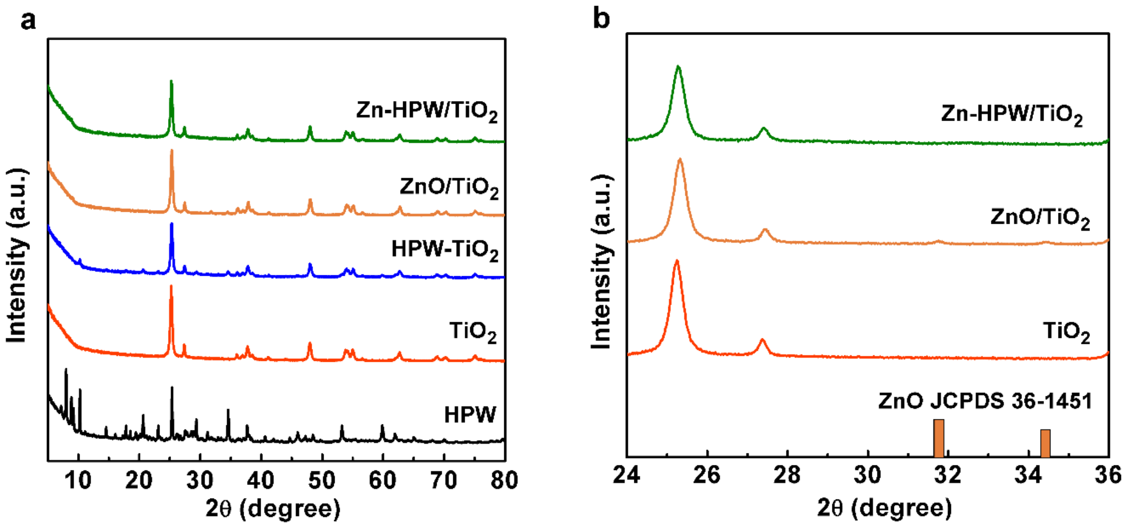

Supplementary Figure 3. XRD patterns of different nanocomposites catalysts. (**a**) 2 θ range: 5-80° and (**b**) 2 θ range 24-36°.

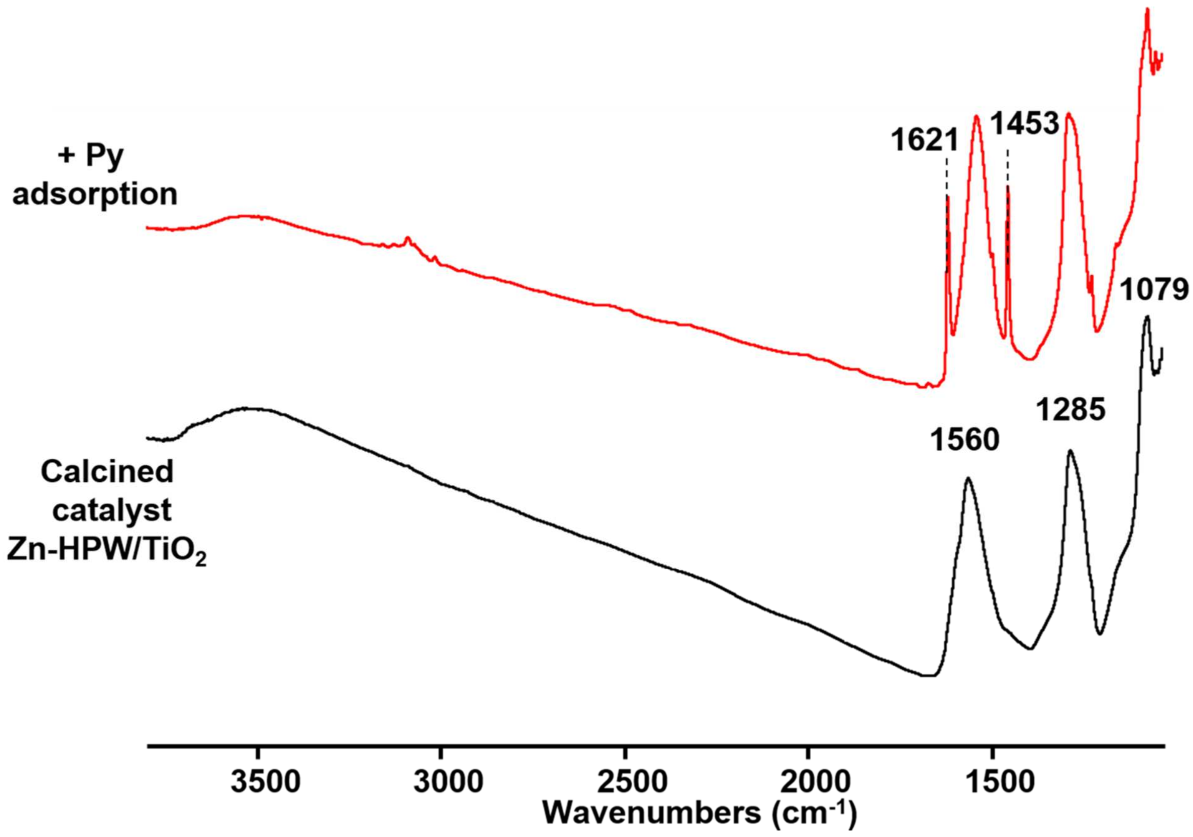

Supplementary Figure 4. FTIR spectra of the Zn-HPW/TiO_2 catalyst before and after adsorption of pyridine and sample evacuation at 200°C.

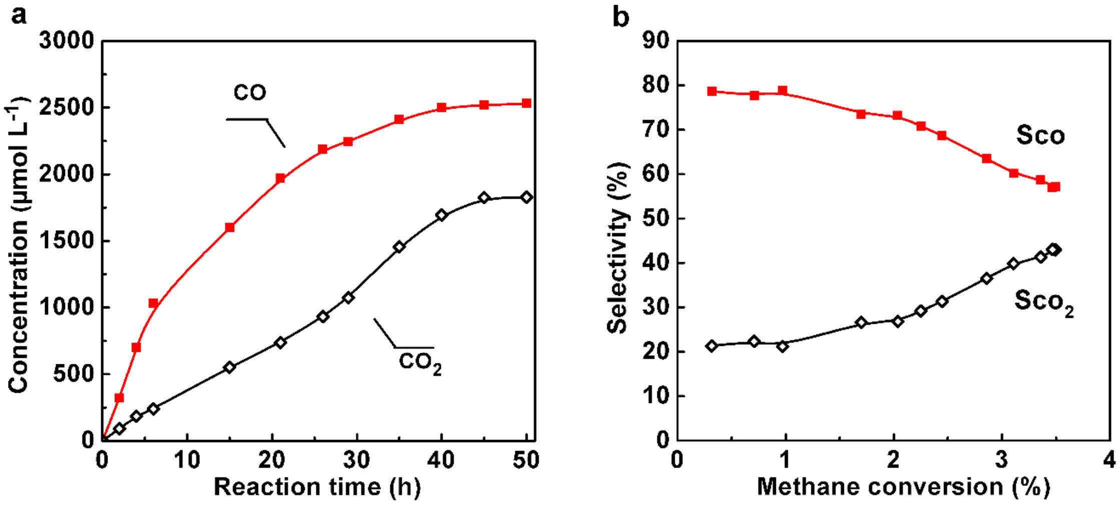

Supplementary Figure 5. (**a**). CO and CO_2 concentrations in the reactor as function of time on stream on Zn-HPW-TiO_2 catalyst; (**b**) CO and CO_2 selectivities as functions of methane conversion. Reaction conditions: catalyst, 0.1 g; Gas phase pressure, CH_4 0.3 MPa, Air 0.1 MPa; irradiation time, 50h.

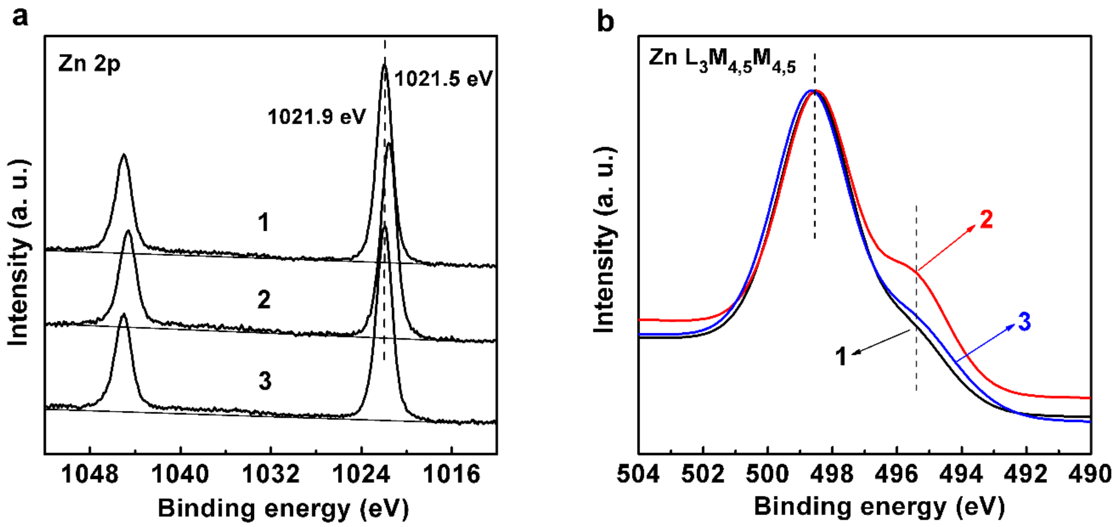

Supplementary Figure 6. XPS (**a**) and Zn $L_3M_{4.5}M_{4.5}$ Auger spectra, (**b**) of Zn-HPW/TiO_2 catalysts in the regions of Zn 2p. (1). fresh catalyst, (2). treatment in 0.3 MPa CH_4 under 400 W Xe lamp for 12h, (3). regeneration in 0.1 MPa air under 400 W Xe lamp for 12h.

The positions of all Auger peaks were aligned by the C 1s peak, and a Shirley background was subtracted. The Zn Auger peak from Zn-HPW/TiO_2 catalysts in **Supplementary Figure 6b** is normalized to the peak height of the ZnO Auger feature.

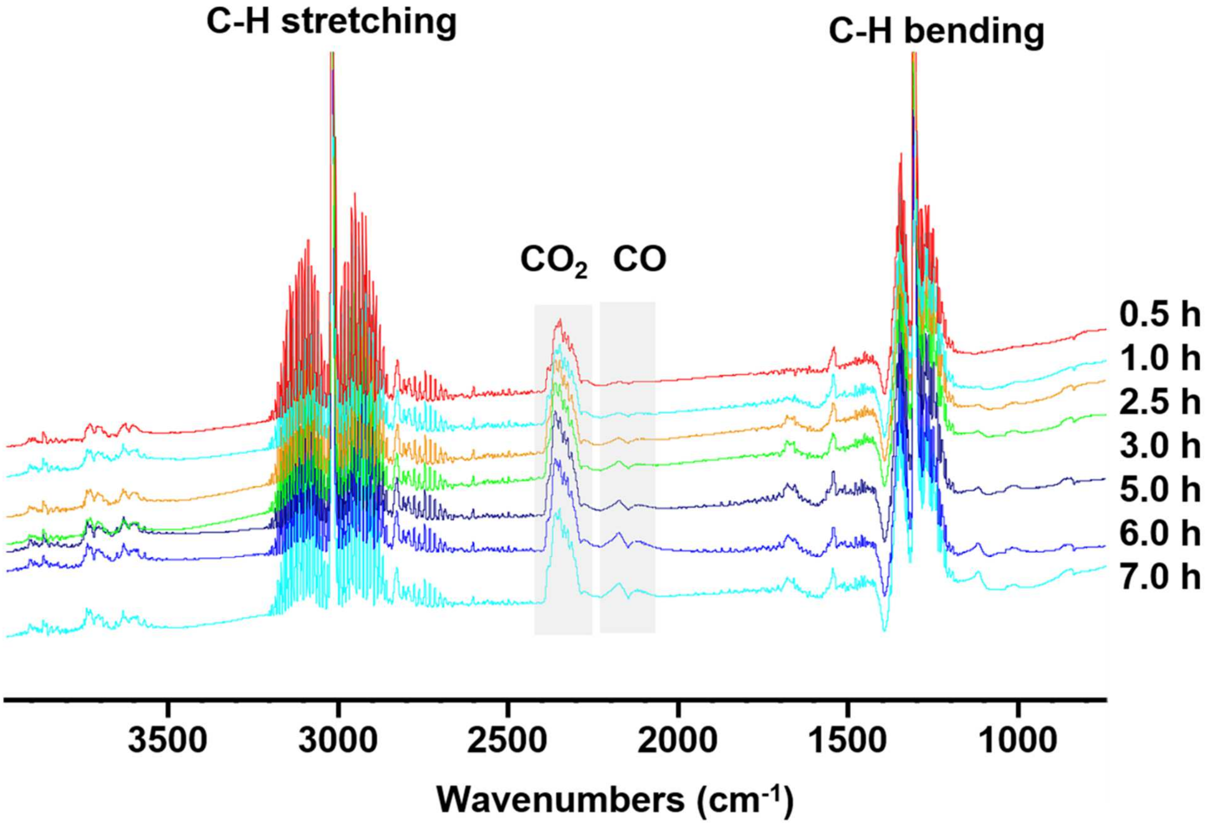

Supplementary Figure 7. FTIR spectra of gaseous phase during methane photocatalytic oxidation over the Zn-HPW/TiO_2 catalyst as a function of reaction time.

IR analysis of the gaseous phase (**Supplementary Figure 7**) clearly shows the presence of methane in the IR cell at the initial periods of the reaction (CH rotation- stretching and rotation-bending bands at around 3020 cm^{-1} and 1300 cm^{-1} respectively). At longer reaction time, gaseous carbon monoxide was identified by rotation- stretching bands at 2150 cm^{-1}. In agreement with the results of photocatalytic tests, the intensity of the carbon monoxide IR band at 2150 cm^{-1} increases with the reaction time.

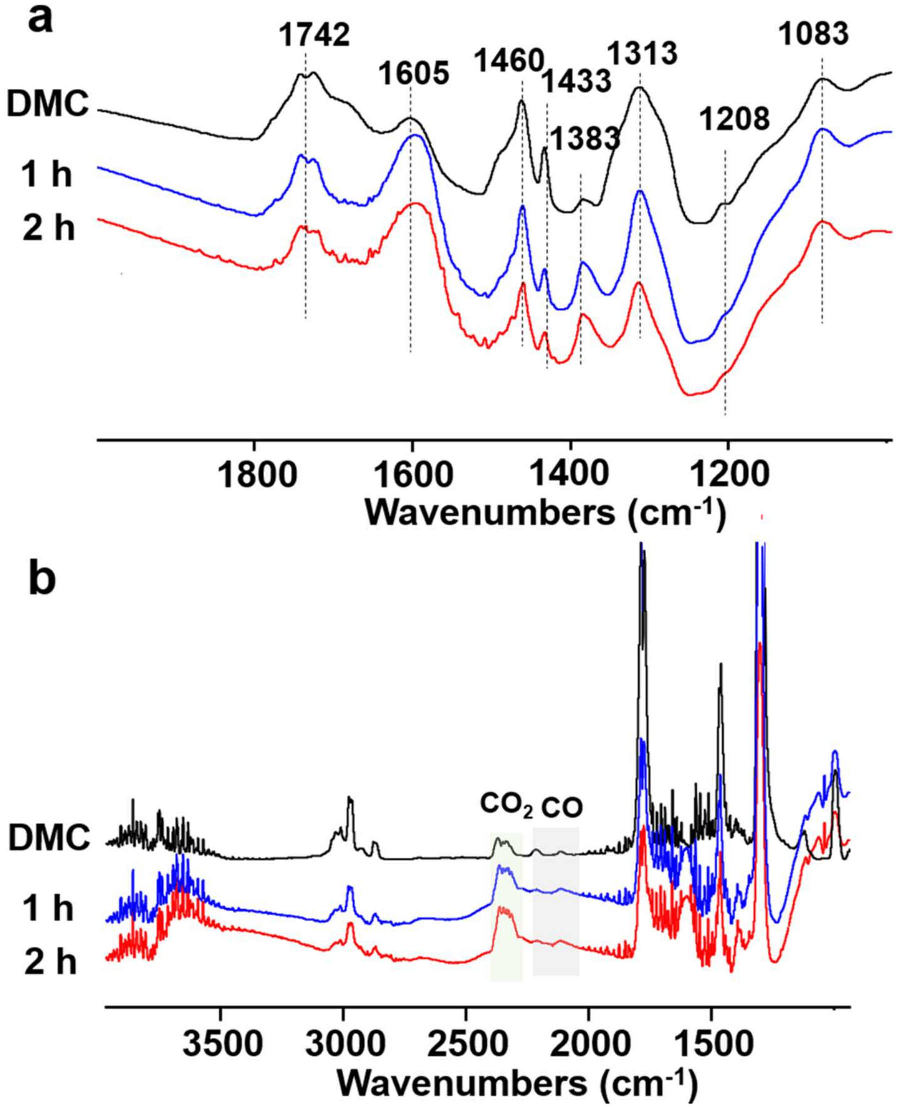

Supplementary Figure 8. IR spectra of adsorbed DMC during exposure to light (**a**) and gas phase analysis (**b**).

(The bands at 1742, 1460, and 1313 cm^{-1} are attributable to C=O stretching, $\nu_{as}(CO_3)$ and $\nu_s(CO_3)$ of O–C–O stretching modes of carbonate species associated with the Zn cations.

This assignment is supported by the work of DMC adsorption over cationic zeolites [*T. Beutel, J. Chem. Soc., Faraday Trans., 1998,94, 985-993; Y. Zhang, A. T. Bell, Journal of Catalysis 255 (2008) 153–161*]. The bands at 1208 and 1083 cm^{-1} might be assigned to ν(C-O) stretching bands in carbonate and methoxy groups, respectively.)

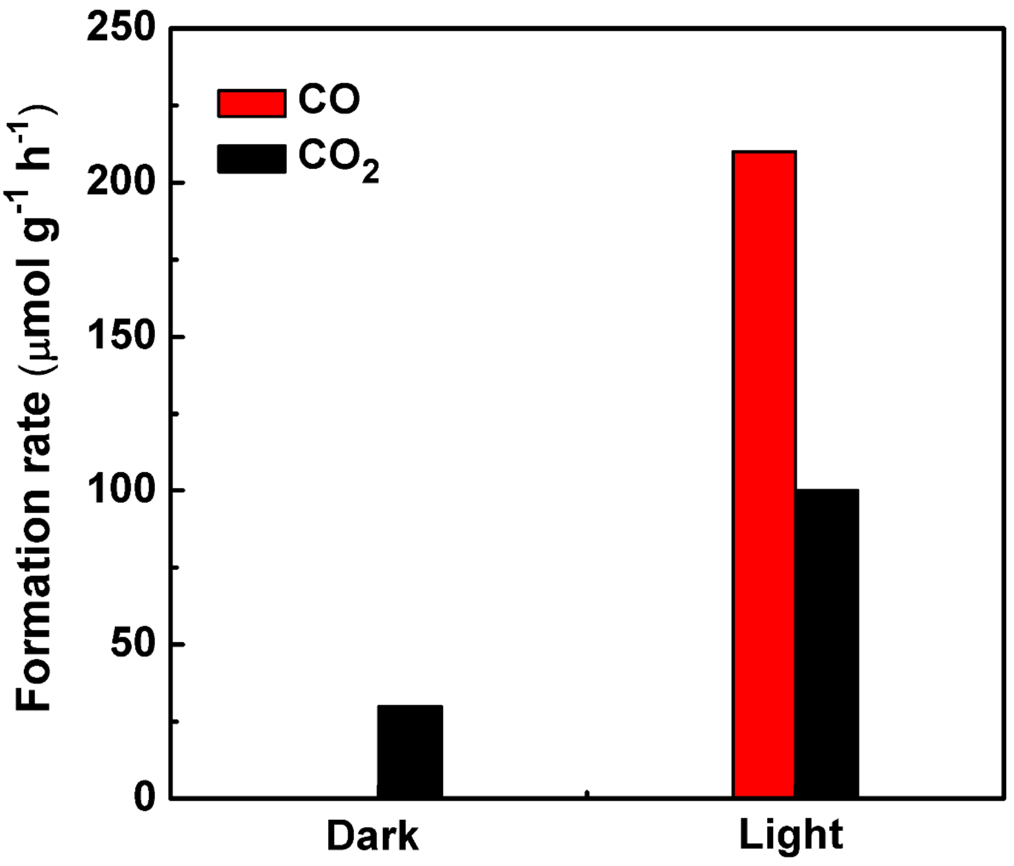

Supplementary Figure 9. Rates of production of CO and CO_2 during decomposition of DMC over the Zn-HPW/TiO_2 catalyst .Reaction condition: Catalyst 0.1 g, Ar 0.2 MPa; DMC 2 mL; 6 h in dark or under light.

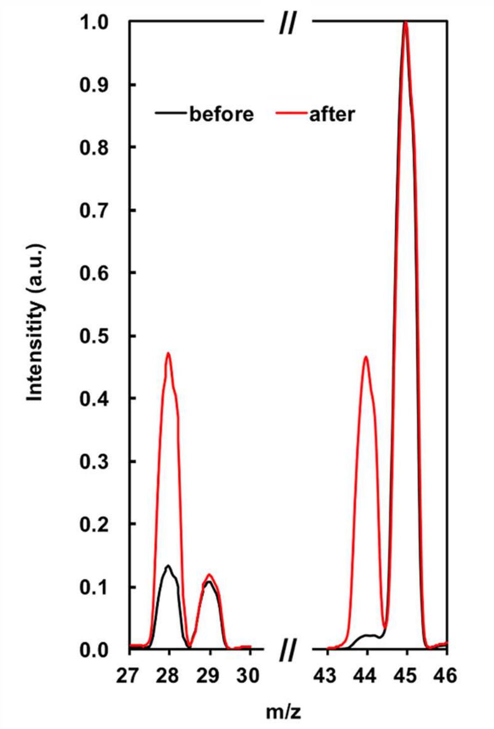

Supplementary Figure 10. Mass spectrum in isotopic $^{13}CO_2$ labeling experiments (normalized); black = before and red = after photocatalytic reaction.

Supplementary Figure 10 presents mass spectrum recorded before and after photocatalytic reaction. The experiments were conducted under a $^{12}CH_4$, O_2 and $^{13}CO_2$ atmosphere (0.3 MPa of CH_4, 0.1 MPa of O_2 and 1% isotopic $^{13}CO_2$).

To facilitate reading, signals have been normalized to the maximum intensity of peak m/z=45. Before reaction (black curve), m/z=45 corresponding to $^{13}CO_2$ is clearly visible together with signal at m/z=29 corresponding to $^{13}CO_2$ cracking to $^{13}CO^+$ fragments in the mass spectrometer ion source. Peaks at m/z=28 represents residual N_2 in the mass spectrometer vacuum chamber or CO species generated by the ion source. m/z=44 represents residual CO_2 in the vacuum chamber and $^{12}CO_2$ impurity contained in the $^{13}CO_2$ cylinder. After reaction (red curve), m/z=28 and m/z=44 signals increase due to the production of ^{12}CO and $^{12}CO_2$ from the photocatalytic oxidation of methane. m/z=29 signal increases due to the production of ^{13}CO. Isotope labelling therefore suggests that some of added $^{13}CO_2$ is converted to ^{13}CO.

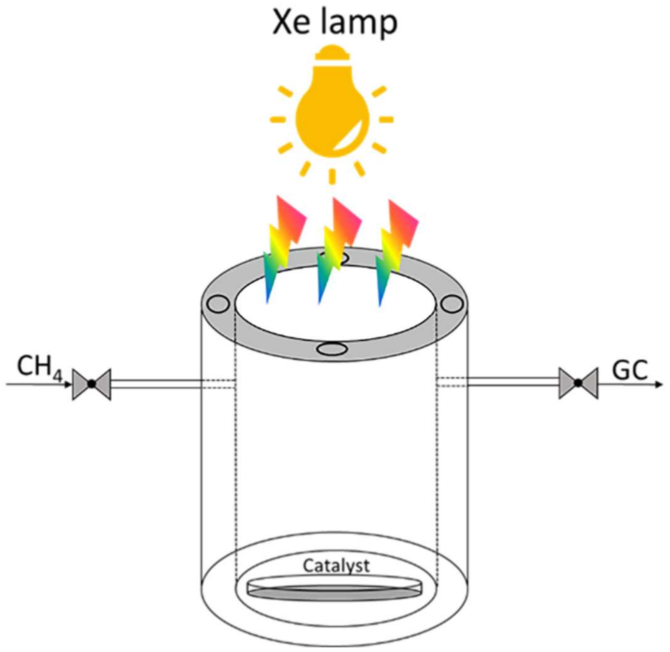

Supplementary Figure 11. Schema of photocatalytic reactor.
